# Supplementary figures and images for: Basement membrane diversification relies on two competitive secretory routes defined by Rab10 and Rab8 and modulated by dystrophin and the exocyst complex
Source: PLoS Genet. 2024 Mar 4;20(3):e1011169. doi: 10.1371/journal.pgen.1011169 (PMC10939200; doi:10.1371/journal.pgen.1011169)

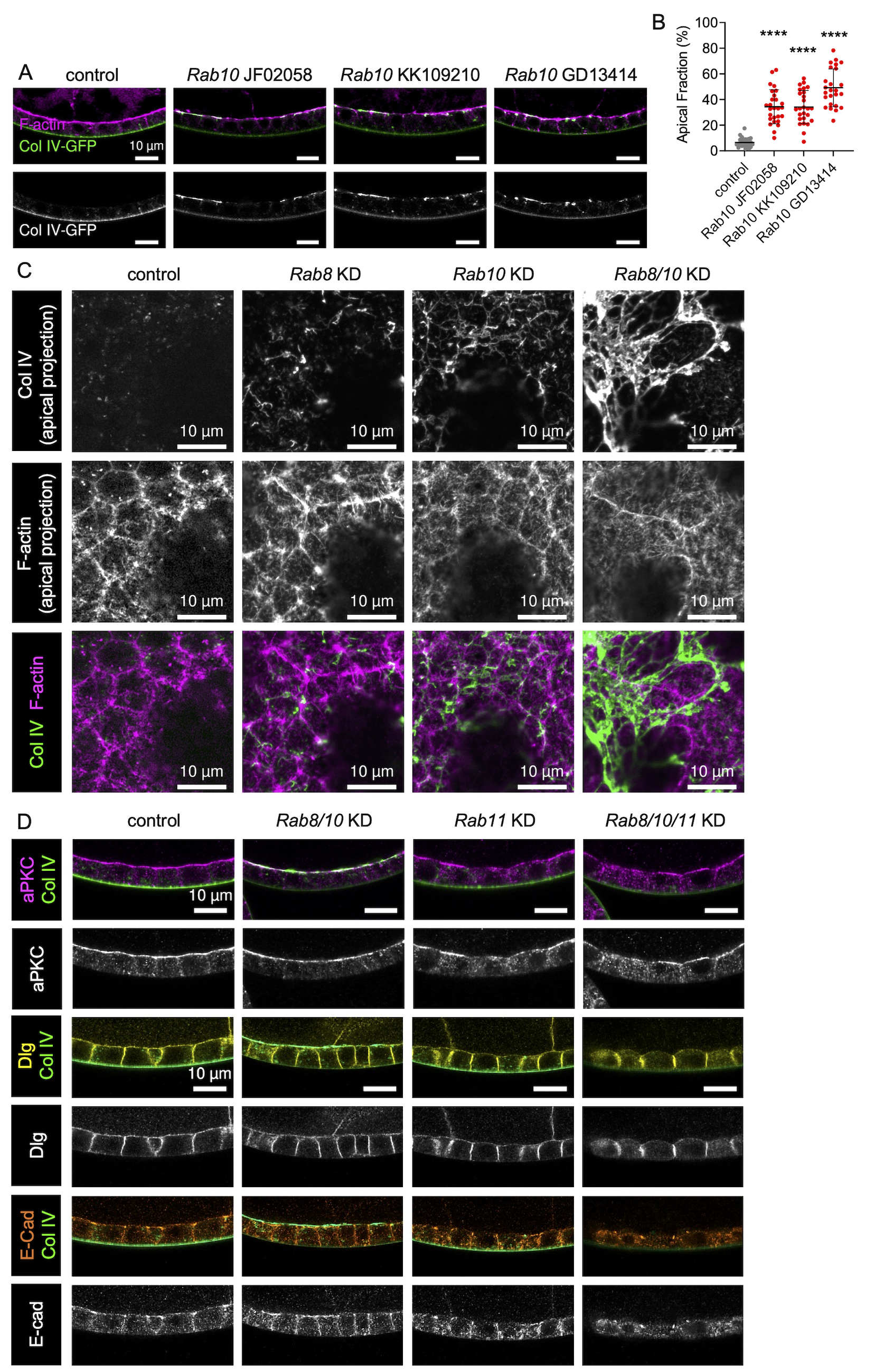

Supplement: S1 Fig — (A) Cross-sections of stage 8 ovarian follicles showing Col IV-GFP (green, top; white, bottom) and F-actin (magenta) localization in control line (left) or tj>Rab10 RNAi (Rab10 knock-down) indicated lines: JF02058, KK109210 or GD13414. (B) Apical fraction quantification (%) of Col IV-GFP fluorescence intensity in stage 8 follicles of the indicated genotypes. Data are the mean ± SD; ****p <0.0001 (Ordinary one-way ANOVA with Tukey’s comparison test); n = 14, 14, 13, 14 follicles. (C) Projections of the apical region of stage 8 follicles that capture some of the apical surface due to the tissue curvature showing the ectopic Col IV-GFP (white, top; green, bottom) and F-actin (white, middle; magenta, bottom) in follicular epithelia of the indicated genotypes: control, Rab8 KD, Rab10 KD and Rab8-Rab10 double KD. (D) Cross-sections of stage 8 follicles of the indicated genotypes (control, Rab8-Rab10 double KD, Rab11 KD, Rab8-Rab10-Rab11 triple KD, showing aPKC (magenta, 1st line; white, 2nd line), Dlg (yellow, 3rd line; white, 4th line), E-Cad (orange, 5th line; white, 6th line) and Col IV-GFP (green). (TIFF) [file pgen.1011169.s001.tiff]

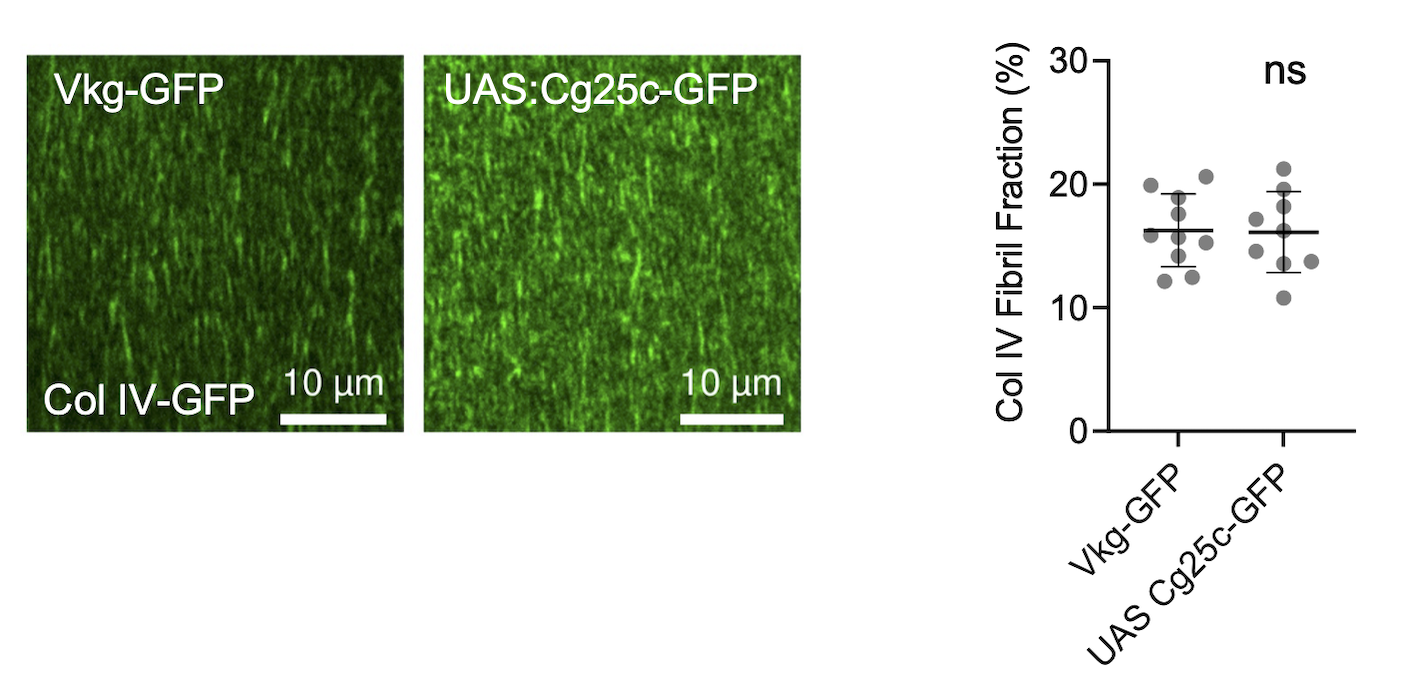

Supplement: S2 Fig — Quantifications of BM fibril fraction (%) in stage 8 follicles of the indicated genotypes. Data are the mean ± SD; ns, not significant (unpaired t test). In order on the graph, n = 10, 9 follicles. (TIFF) [file pgen.1011169.s002.tiff]

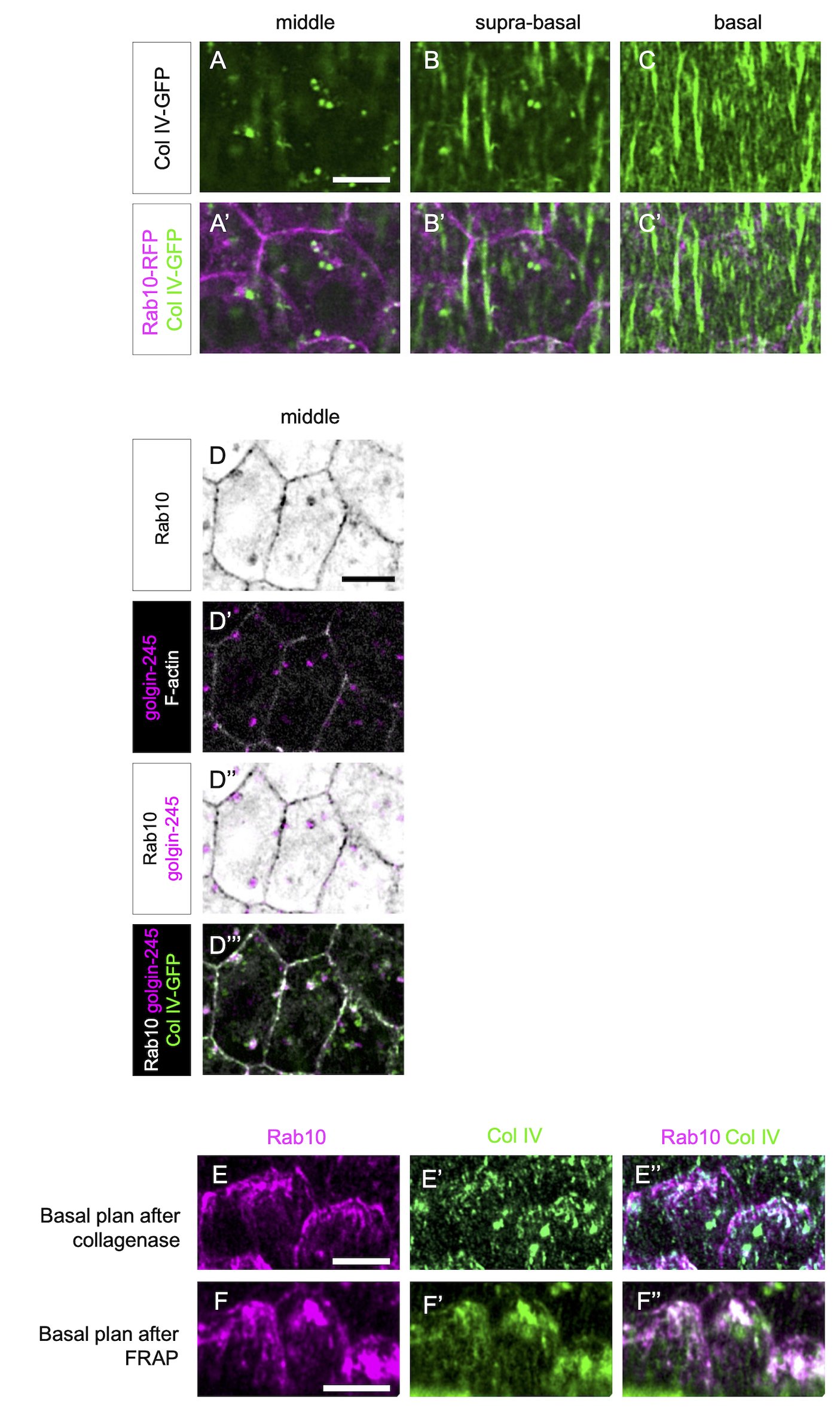

Supplement: S3 Fig — (A-C’) Basal (C, C’), suprabasal (i.e. 0.4 μm above the basal surface) (B, B’) and middle (i.e. 0.8 μm below basal surface) (A, A’) views of stage 8 follicles that overexpress RFP-tagged Rab10 (magenta) and endogenous Col IV-GFP (green). (D-D”’) Middle view of stage 8 follicles that overexpress RFP-tagged Rab10 (black in 1st and 3rd line, white in 4th line), express endogenous Col IV-GFP (green) and that are stained with anti-golgin-245 antibody (magenta) and F-actin (white in 2nd line). (E-E”) Basal view of stage 8 follicles treated with collagenase before fixation to visualize intracellular endogenous basal Col IV-GFP (green) and Rab10-RFP (magenta). (F-F”) Basal view of a photobleached window at the surface of stage 8 living follicles taken 20 minutes after photobleaching to visualize intracellular basal Rab10-RFP (magenta) and endogenous Col IV-GFP (green) expression in non-bleached cells that have migrated in the bleached BM area. Scale bars, 5 μm. (TIFF) [file pgen.1011169.s003.tiff]

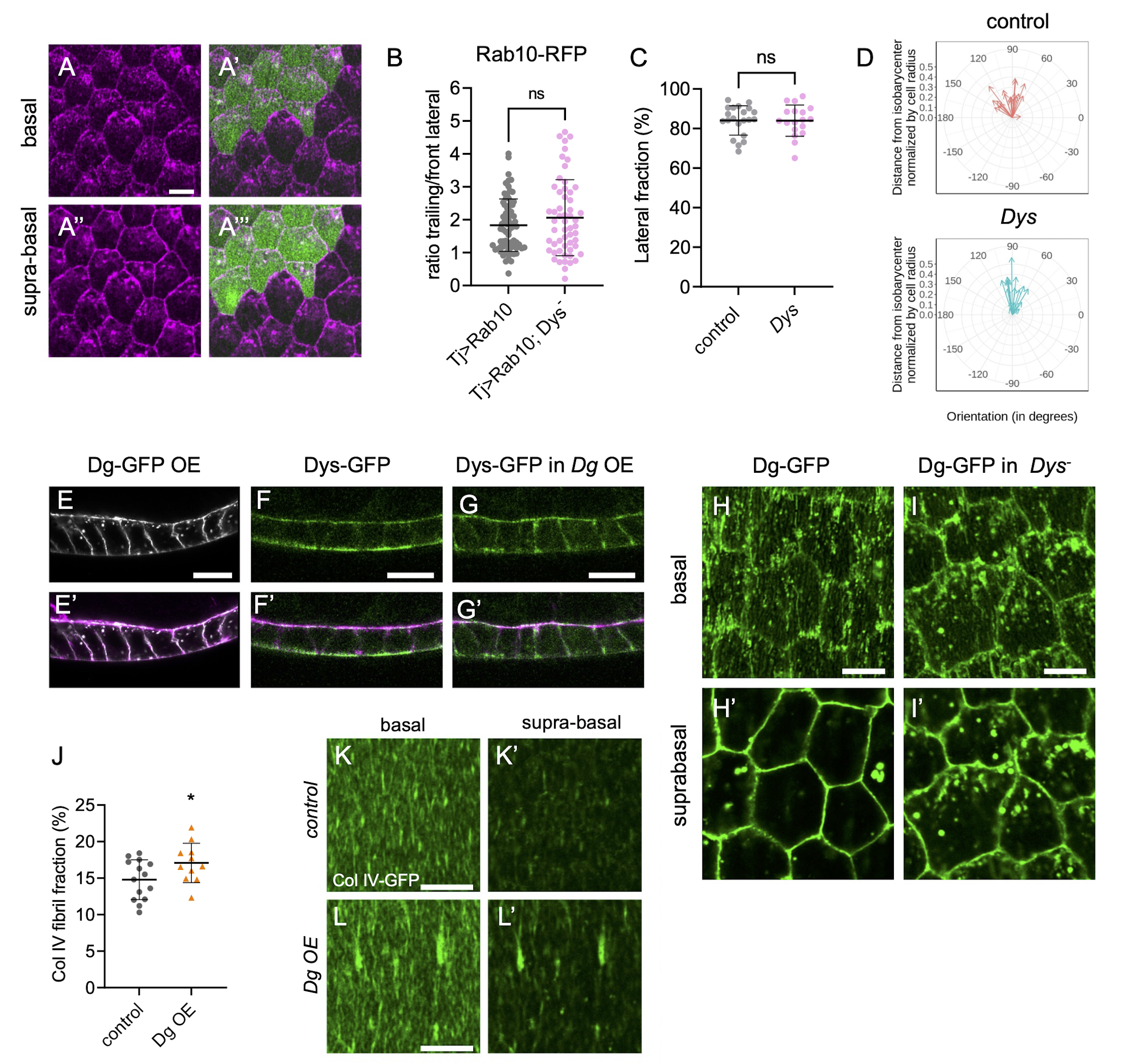

Supplement: S4 Fig — (A-A‘) Basal and (A”,A”’) suprabasal view of stage 8 follicles expressing Rab10-RFP (magenta) in WT cells (GFP cells, green) or in Dys null mutant cell clones (no GFP). (B) Rab10-RFP mean signal intensity at the trailing edge vs adjacent lateral front side of follicle cells of the indicated genotypes, on 0.45 μm projections starting 0.15 μm below the basal surface. Data are the mean ± SD. In order on graph, n = 73 and 56 follicle cells. (C) Quantification of the lateral fraction of BM secretion (%) in single-cell clones overexpressing Col IV-GFP in control and Dys null mutant follicles. Data are the mean ± SD. In order on graph, n = 21 and 18 cells. ns, not significant (unpaired t test). (D) Quantification of the planar orientation of lateral BM protein secretion from individual single-cell clones in control (red) and Dys null mutant (blue). (E-G) Sagittal view of follicles overexpressing Dg-GFP (E, E’) or expressing endogenous Dys-sfGFP in WT context (F, F’) or in Dg overexpression (G-G’). Dg-GFP is shown in white, Dys-sfGFP in green and F-actin in magenta. (H, I) basal and (H’, I’) suprabasal images of tj>Dg-GFP in (H) WT or (I) Dys mutant conditions. (J) Quantifications of BM fibril fraction (%) of stage 8 WT or tj> Dg follicles. Data represent mean ± SD. Unpaired t test, *p < 0.05. In order on graph, n = 14 and 11 follicles. (K-L) Basal and (K’, L’) suprabasal views of the BM on stage 8 control (K, K’) and tj> Dg (L, L’)) follicles visualized with Col IV-GFP (green). Scale bars, 5 μm in (A, H-I’), 10μm in (E-G’ and K-L’) (TIFF) [file pgen.1011169.s004.tiff]

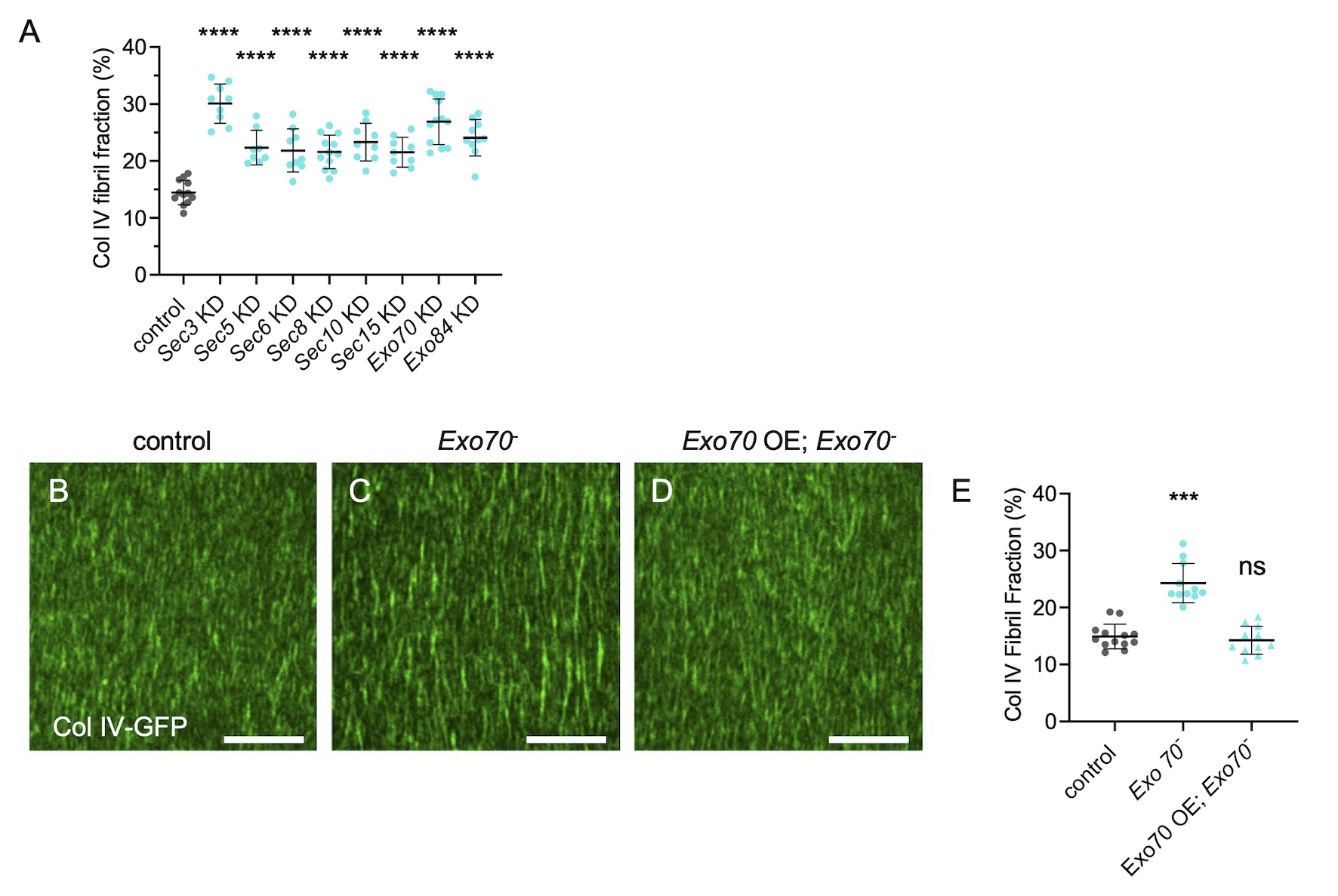

Supplement: S5 Fig — (A) Quantifications of BM fibril fraction (%) in stage 8 follicles of the indicated genotypes. In order on the graph, n = 12, 9, 8, 9, 12, 9, 9, 12 and 10 follicles. (B-D) Basal view of the BM visualized with Col IV-GFP at stage 8 in (B) control, (C) Exo70 null mutant, (D) Exo70 null mutant with Exo70 OE follicles. Scale bars, 10 μm. (E) Quantification of BM fibril fraction (%) in stage 8 follicles of the indicated genotypes. In order on the graph, n = 13, 11, 11 follicles. For all graphs, data are the mean ± SD; ***p <0.01, ****p <0.0001 (Ordinary one-way ANOVA with Dunnett’s multiple comparisons test in A, and Kruskal-Wallis with Dunn’s comparisons test in E). (TIFF) [file pgen.1011169.s005.tiff]
