# Supplementary material for: Basement membrane diversification relies on two competitive secretory routes defined by Rab10 and Rab8 and modulated by dystrophin and the exocyst complex
Source: PLoS Genet. 2024 Mar 4;20(3):e1011169. doi: 10.1371/journal.pgen.1011169 (PMC10939200; doi:10.1371/journal.pgen.1011169)
Supplement: S1 Table — (DOCX) [file pgen.1011169.s006.docx]

**S1 Table: Resources and reagents**

| RESOURCE OR REAGENT | SOURCE | IDENTIFIER |
| --- | --- | --- |
| **Experimental models: Organisms/Strains** | | |
| tj:Gal4 : P{w+mW.hs=GawB}NP1624 | Kyoto Stock Center  (DGRC) | FlyBase ID:  FBti0034540;  DGRC: 104055 |
| colIV-GFP : P{PTT-un1} vkg G205 | Kyoto Stock Center (DGRC) | FlyBase ID:  FBti0074199; DGRC: 110626 |
| tub:Gal80ts : P{w[+mC]=tubP-GAL80[ts]}20 | Bloomington Drosophila Stock Center | FlyBase ID:  FBti0027796;  BDSC: 67059 |
| Rab10 RNAi : UAS:Rab10(JF02058) | Bloomington Drosophila Stock Center | FlyBase ID:  FBal0220549;  BDSC: 26289 |
| Rab10 RNAi : UAS:Rab10(KK109210) | Vienna Drosophila Resource Center | FlyBase ID:  FBal0236140;  VDRC: 101454 |
| Rab10 RNAi : UAS:Rab10(GD13414) | Vienna Drosophila Resource Center | FlyBase ID:  FBal0208199;  VDRC: 28758 |
| Rab8 RNAi : UAS:Rab8(GD12520) | Vienna Drosophila Resource Center | FlyBase ID:  FBal0208212;  VDRC: 28092 |
| Rab11 RNAi : UAS:Rab11(KK108297) | Vienna Drosophila Resource Center | FlyBase ID:  FBal0235482;  VDRC: 108382 |
| UAS:Rab10-RFP (chr2) | Horne- Badovinac lab [1] |  |
| UAS:Rab10-RFP (chr3) | Horne- Badovinac lab [1] |  |
| UAS:Rab8-YFP : UASp-YFP.Rab8}45 | Bloomington Drosophila Stock Center | FlyBase ID:  FBti0100806;  BDSC: 9782 |
| UAS:LacZ : P{UAS-lacZ.NZ}J312 | Bloomington Drosophila Stock Center | FlyBase ID:  FBti0012289  BDSC: 3956 |
| UAS:Cg25c-GFP | V. Van De Bor gift | FlyBase ID:  FBal0336357 |
| Rab10-eYFP ki : TI [56]Rab10[EYFP] | Bloomington Drosophila Stock Center | FlyBase ID:  FBti0178272;  BDSC: 62548 |
| Dys::sfGFP::LoxP | this study |  |
| UAS:Dg-GFP | Marie Laure Parmentier gift | FlyBase ID:  FBal0244402 |
| UAS:Dg | Ruhola-Baker lab [2] | FlyBase ID: FBal0145088 |
| Dys^E17^ | Ray lab [3] | FlyBase ID: FBal0241311 |
| DysExel6184: FRT 82B, Df(3R)Exel6184 | Bloomington Drosophila Stock Center | FlyBase ID: FBab0038239;  BDSC: 7663 |
| y,w, hs:FLP : P{ry[+t7.2]=hsFLP}22, w[*] | Bloomington Drosophila Stock Center | FlyBase ID: FBst0008862; BDSC: 8862 |
| y,w, hs:FLP : P{ry[+t7.2]=hsFLP}1, w[1118] | Bloomington Drosophila Stock Center | FlyBase ID: FBti0002044 or BDSC: 6 |
| FRT82B | Bloomington Drosophila Stock Center | FlyBase ID: FBti0002074;  BDSC: 2035 |
| P{y[+t7.7] w[+mC]=CoinFLP-GAL4}attP | Bloomington Drosophila Stock Center | FlyBase ID: FBtp0096005; BDSC: 58750 |
| Exo70^MB04553^ | Bloomington Drosophila Stock Center | FlyBase ID:  FBti0078291;  BDSC: 24721 |
| Df(3L)BSC815 | Bloomington Drosophila Stock Center | FlyBase ID:  FBab0045927;  BDSC: 27576 |
| Exo70 RNAi : Exo70(KK101154) | Vienna Drosophila Resource Center | FlyBase ID:  FBal0231092;  VDRC: 103717 |
| Exo70 RNAi : Exo70(GD12140) | Vienna Drosophila Resource Center | FlyBase ID:  FBal0209535;  VDRC: 27867 |
| Sec3 RNAi : Sec3(GD10687) | Vienna Drosophila Resource Center | FlyBase ID:  FBal0210549;  VDRC: 35806 |
| Sec5 RNAi : Sec5(GLC01676) | Bloomington Drosophila Stock Center | FlyBase ID:  FBal0291964;  BDSC: 50556 |
| Sec6 RNAi : Sec6(KK102544) | Vienna Drosophila Resource Center | FlyBase ID:  FBal0231852;  VDRC: 105836 |
| Sec8 RNAi : Sec8 (GD8828) | Vienna Drosophila Resource Center | FlyBase ID:  FBal0210555;  VDRC: 45032 |
| Sec10 RNAi : Sec10(JF02633) | Bloomington Drosophila Stock Center | FlyBase ID:  FBal0242222;  BDSC: 27483 |
| Sec15 RNAi : Sec15(GD12109) | Vienna Drosophila Resource Center | FlyBase ID:  FBal0210546;  VDRC: 35161 |
| Exo84 RNAi : Exo84(KK100045) | Vienna Drosophila Resource Center | FlyBase ID:  FBal0230555;  VDRC: 108650 |
| UAS: YFP Rab8 (chr 3) | Bloomington | Bl#23272 |
| UAS: Scarlet-Exo70 | this study |  |
| **Antibodies** | | |
| anti-GFP, goat | Abcam | 5450; dilution 1/1000 |
| anti-aPKc, goat | Santa Cruz Biotechnology | Sc-216-g; dilution 1/500 |
| anti-Dlg, mouse | DSHB | 4F3; dilution 1/100 |
| anti-E-Cad, rat | DSHB | DCAD2; dilution 1/100 |
| Anti-golgin-245, goat | DSHB | Golgin245; dilution 1/1000 |
| anti-goat-Cy5, Donkey | Jackson Immunoresearch | 705-175-147; dilution 1/1000 |
| Anti-goat-Cy3, Donkey | Jackson Immunoresearch | 705-165-147; dilution 1/1000 |
| **Chemicals, Peptides, and Recombinant Proteins** | | |
| Phalloidin Atto-488, -550, -633 | Sigma | #49409, #19083, #68825 |
| Collagenase | Worthington Biochemical Corporation | # LS005273 |
| **Software and Algorithms** | | |
| Fiji |  |  |
| R |  |  |
| Prism |  |  |

References :

1. Isabella AJ, Horne-Badovinac S. Rab10-Mediated secretion synergizes with tissue movement to build a polarized basement membrane architecture for organ morphogenesis. Dev Cell 2016, Jul 11;38(1):47-60.

2. Deng WM, Schneider M, Frock R, Castillejo-Lopez C, Gaman EA, Baumgartner S, Ruohola-Baker H. Dystroglycan is required for polarizing the epithelial cells and the oocyte in drosophila. Development 2003, Jan;130(1):173-84.

3. Christoforou CP, Greer CE, Challoner BR, Charizanos D, Ray RP. The detached locus encodes drosophila dystrophin, which acts with other components of the dystrophin associated protein complex to influence intercellular signalling in developing wing veins. Dev Biol 2008, Jan 15;313(2):519-32.
