# Supplementary material for: Basement membrane diversification relies on two competitive secretory routes defined by Rab10 and Rab8 and modulated by dystrophin and the exocyst complex
Source: PLoS Genet. 2024 Mar 4;20(3):e1011169. doi: 10.1371/journal.pgen.1011169 (PMC10939200; doi:10.1371/journal.pgen.1011169)
Supplement: S2 Table — (h: hours; HS: heat-shock). (DOCX) [file pgen.1011169.s007.docx]

**S2 Table: Genotypes and specific conditions**

(h: hours; HS: heat-shock)

| **Figure** | **Genotype** | **Condition** |
| --- | --- | --- |
| Fig 1 B-I | *tj:Gal4, colIV-GFP, tub:Gal80^ts^ / CyO*  *tj:Gal4, colIV-GFP, tub:Gal80^ts^ / UAS:Rab8 RNAi*  *tj:Gal4, colIV-GFP, tub:Gal80^ts^ /+; UAS:Rab10 RNAi /+*  *tj:Gal4, colIV-GFP, tub:Gal80^ts^ / UAS:Rab8 RNAi; UAS:Rab10 RNAi /+*  *tj:Gal4, colIV-GFP, tub:Gal80^ts^ / UAS:Rab11 RNAi*  *tj:Gal4, colIV-GFP, tub:Gal80^ts^ / UAS:Rab8 RNAi, UAS:Rab11 RNAi; UAS:Rab10 RNAi /+* | 18°C, 48h 30°C |
| Fig S1 A-B  C  D | *tj:Gal4, colIV-GFP / CyO*  *tj:Gal4, colIV-GFP /+; UAS:Rab10 RNAi JF02058/+*  *tj:Gal4, colIV-GFP / UAS:Rab10 RNAi KK109210*  *tj:Gal4, colIV-GFP / UAS:Rab10 RNAi GD13414*  *tj:Gal4, colIV-GFP, tub:Gal80^ts^ / CyO*  *tj:Gal4, colIV-GFP, tub:Gal80^ts^ / UAS:Rab8 RNAi*  *tj:Gal4, colIV-GFP, tub:Gal80^ts^ /+; UAS:Rab10 RNAi /+*  *tj:Gal4, colIV-GFP, tub:Gal80^ts^ / UAS:Rab8 RNAi; UAS:Rab10 RNAi /+*  *tj:Gal4, colIV-GFP, tub:Gal80^ts^ / CyO*  *tj:Gal4, colIV-GFP, tub:Gal80^ts^ / UAS:Rab8 RNAi; UAS:Rab10 RNAi /+*  *tj:Gal4, colIV-GFP, tub:Gal80^ts^ / UAS:Rab11 RNAi*  *tj:Gal4, colIV-GFP, tub:Gal80^ts^ / UAS:Rab8 RNAi, UAS:Rab11 RNAi; UAS:Rab10 RNAi /+* | 30°C  18°C, 48h 30°C  18°C, 48h 30°C |
| Fig 2 B-H  I | *tj:Gal4, colIV-GFP / CyO*  *tj:Gal4, colIV-GFP /+; UAS:Rab10-RFP /+*  *tj:Gal4, colIV-GFP /+; UAS:Rab10 RNAi /+*  *tj:Gal4, colIV-GFP / UAS:Rab8-YFP*  *tj:Gal4, colIV-GFP / UAS:Rab8 RNAi*  *tj:Gal4, colIV-GFP / UAS: Rab8-YFP; UAS:Rab10-RFP /+*  *tj:Gal4, colIV-GFP / +; UAS:LacZ /+*  *tj:Gal4, colIV-GFP/ UAS:Rab8-YFP; UAS:LacZ /+*  *tj:Gal4, colIV-GFP /+; UAS:LacZ / UAS:Rab10-RFP*  *tj:Gal4, colIV-GFP / UAS:Rab8-YFP; UAS:Rab10-RFP /+* | 30°C  30°C |
| Fig 3 B-J | *hs:flp1; Act5c CoinFLP-Gal4/UAS:colIV-GFP*  *hs:flp1; Act5c CoinFLP-Gal4/UAS:colIV-GFP UAS:Rab10-RFP /+*  *hs:flp1; Act5c CoinFLP-Gal4/UAS:colIV-GFP UAS:Rab8-YFP /+* | 25°C, HS 15 min 37°C, 30°C 9h |
| Fig S2 | *tj:Gal4, colIV-GFP / CyO*  *tj:Gal4 / UAS:Cg25c-GFP* | 25°C |
| Fig 4 A-D  E-H  I  J-K | *Rab10-eYFP ki*  *tj:Gal4, colIV-GFP /+; UAS:Rab10-RFP /+*  *Dys-sfGFP*  *tj:Gal4 /+; UAS:Rab10-RFP / Dys-sfGFP* | 25°C  25°C  25°C  25°C |
| Fig S3 | *tj:Gal4, colIV-GFP /+; UAS:Rab10-RFP /+* | 25°C |
| Fig 5 A-L  M  N  O  P  Q  R  S  T-U | *tj:Gal4, colIV-GFP /+; Dys^E17^ / Dys^Exel6184^*  *tj:Gal4, colIV-GFP / UAS:Rab10-RFP*  *tj:Gal4, colIV-GFP / UAS:Rab10-RFP; Dys^E17^ / Dys^Exel6184^*  *tj:Gal4, colIV-GFP / UAS:Rab8 RNAi*  *tj:Gal4, colIV-GFP / UAS:Rab8 RNAi; Dys^E17^ / Dys^Exel6184^*  *tj:Gal4, colIV-GFP / UAS:Rab10-RNAi*  *tj:Gal4, colIV-GFP / UAS:Rab10 RNAi; Dys^E17^ / Dys^Exel6184^*  *tj:Gal4 / UAS: Dg-GFP*  *tj:Gal4 / UAS:Dg; Dys-sfGFP /+*  *tj:Gal4 / UAS:Dg-GFP; UAS:Rab10-RFP /+*  *tj:Gal4 /UAS:Dg-GFP; UAS:Rab10-RFP, Dys^E17^/Dys^Exel6184^*  *tj:Gal4 / UAS: Dg-GFP*  *tj:Gal4 / UAS:Dg-GFP; UAS:Rab10-RFP /+*  *tj:Gal4 /UAS:Dg-GFP; UAS:Rab10-RFP, Dys^E17^/Dys^Exel6184^*  *Dys-sfGFP*  *tj:Gal4 / UAS:Dg; Dys-sfGFP /+*  *tj:Gal4 /+; UAS:Rab10-RFP /+*  *tj:Gal4 / UAS:Dg-GFP; UAS:Rab10-RFP /+*  *tj:Gal4 /UAS:Dg-GFP; UAS:Rab10-RFP, Dys^E17^/Dys^Exel6184^*  *tj:Gal4, colIV-GFP /+*  *tj:Gal4, colIV-GFP / UAS:Dg* | 25°C, 30°C 72h  25°C, 30°C 48h  25°C, 30°C 72h  25°C, 30°C 72h  25°C, 30°C 72h  25°C, 30°C 72h  25°C, 30°C 72h  25°C, 30°C 72h  25°C, 30°C 72h |
| Fig S4 A-B  C-D  E-G  H-I  J-L | *y,w,hs:flp122; tjGal4 / UAS:Rab10-RFP; FRT82B-GFP / FRT82B, Dys^Exel6184^*  *hs:flp1; Act5c CoinFLP-Gal4/UAS:colIV-GFP*  *hs:flp1; Act5c CoinFLP-Gal4/UAS:colIV-GFP; Dys^E17^/Dys^Exel6184^*  *tj:Gal4 / UAS: Dg-GFP*  *Dys-sfGFP*  *tj:Gal4 / UAS:Dg; Dys-sfGFP /+*  *tj:Gal4 / UAS:Dg-GFP*  *tj:Gal4 /UAS:Dg-GFP; Dys^E17^/Dys^Exel6184^*  *tj:Gal4, colIV-GFP /+*  *tj:Gal4, colIV-GFP / UAS:Dg* | 25°C, 2HS 1h, dissection 72h after HS  25°C, HS 15 min 37°C, 30°C 9h  25°C, 30°C 72h  25°C, 30°C 72h  25°C, 30°C 72h |
| Fig 6 C-J  L-P | *Dys-sfGFP /+*  *tj:Gal4 / UAS: Scarlet-Exo70; Dys-sfGFP /+*  *Dys-sfGFP, Exo70^MB04553^ / Df(3L)BSC815*  *tj:Gal4, colIV-GFP / UAS:Rab10-YFP*  *tj:Gal4 / UAS: Scarlet-Exo70; UAS:Rab10-YFP / +*  *tj:Gal4, colIV-GFP / UAS:Rab10-RFP*  *tj:Gal4, colIV-GFP / UAS:Rab10-RFP; Exo70^MB04553^ / Df(3L)BSC815*  *tj:Gal4, colIV-GFP /+*  *tj:Gal4, colIV-GFP /+; Dys^E17^ / Dys^Exel6184^*  *tj:Gal4, colIV-GFP /+; Exo70^MB04553^ / Df(3L)BSC815*  *tj:Gal4, colIV-GFP / UAS:Exo70 RNAi; Dys^E17^ / Dys^Exel6184^* | 25°C  25°C, 30°C 72h  25°C, 30°C 72h  25°C  25°C, 30°C 72h |
| Fig S5 A  B-E | *tj:Gal4, colIV-GFP /+*  *tj:Gal4, colIV-GFP/ UAS:Sec3 RNAi*  *tj:Gal4, colIV-GFP/+; UAS:Sec5 RNAi*  *tj:Gal4, colIV-GFP/ UAS:Sec6 RNAi*  *tj:Gal4, colIV-GFP/ UAS:Sec8 RNAi*  *tj:Gal4, colIV-GFP/+; UAS:Sec10 RNAi*  *tj:Gal4, colIV-GFP/+; UAS:Sec15 RNAi*  *tj:Gal4, colIV-GFP/ UAS:Exo70 RNAi*  *tj:Gal4, colIV-GFP/ UAS:Exo84 RNAi*  *tj:Gal4, colIV-GFP /+*  *tj:Gal4, colIV-GFP /+; Exo70^MB04553^ / Df(3L)BSC815*  *tj:Gal4, colIV-GFP / UAS: Scarlet-Exo70; Exo70^MB04553^ / Df(3L)BSC815* | 25°C, 30°C or  18°C, 30°C 48h  30°C  30°C  25°C  25°C  18°C, 30°C 48h  25°C  30°C  25°C  25°C |
| Fig 7 A-C  D  E-H  I | *hs:flp1; Act5c CoinFLP-Gal4/UAS:colIV-GFP*  *hs:flp1; Act5c CoinFLP-Gal4/UAS:colIV-GFP; Exo70^MB04553^ / Df(3L)BSC815*  *tj:Gal4, colIV-GFP /+; Exo70^MB04553^ / Df(3L)BSC815*  *tj:Gal4, colIV-GFP / UAS:Rab8 RNAi*  *tj:Gal4, colIV-GFP / UAS:Rab8 RNAi; UAS:Exo70 RNAi / +*  *tj:Gal4, colIV-GFP / UAS:Rab10-RFP*  *tj:Gal4, colIV-GFP / UAS:Rab10-RFP; Exo70^MB04553^ / Df(3L)BSC815*  *tj:Gal4, colIV-GFP /+*  *tj:Gal4, colIV-GFP / UAS:Exo70 RNAi*  *tj:Gal4, colIV-GFP /+; UAS:Rab10 RNAi*  *tj:Gal4, colIV-GFP / UAS:Exo70 RNAi; UAS:Rab10 RNAi*  *tj:Gal4, colIV-GFP /+*  *tj:Gal4, colIV-GFP / UAS:Rab8 RNAi* | 25°C, HS 15 min 37°C, 30°C 9h  25°C  25°C, 30°C 72h  25°C, 30°C 72h  25°C  25°C  25°C, 30°C 72h  25°C, 30°C 72h |
