# Supplementary material for: Basement membrane diversification relies on two competitive secretory routes defined by Rab10 and Rab8 and modulated by dystrophin and the exocyst complex
Source: PLoS Genet. 2024 Mar 4;20(3):e1011169. doi: 10.1371/journal.pgen.1011169 (PMC10939200; doi:10.1371/journal.pgen.1011169)
Supplement: Attachment — Submitted filename: full-revision.pdf [file pgen.1011169.s009.pdf]

Full Revision

Manuscript number: RC-2023-01935
Corresponding author(s): Vincent Mirouse

1. General Statements [optional]

First of all, we would like to thank the three reviewers for the interest they expressed in our work. Moreover, we believe that, aided by their suggestions, we managed to significantly improve our manuscript.

2. Point-by-point description of the revisions

From here, Reviewers' comments are in black and our reply in italic dark blue

Reviewer #1 (Evidence, reproducibility and clarity):

This manuscript by Dennis et al. reports a study of the polarized secretion of basement membrane Collagen IV in the Drosophila (fruit fly) follicular epithelium. Using genetic manipulations and confocal imaging, the authors show that Rab-GTPases Rab8 and Rab10, both known to be required for proper basal secretion of Collagen IV (work by the labs of Sally Horne-Badovinac and Trudi Schupbach, respectively), mediate two alternative secretion routes: Rab8 mediates basal-most secretion of soluble Collagen IV that is incorporated homogenously into the basement membrane, whereas Rab10 mediates basal-lateral secretion of Collagen IV that produces insoluble fibers. The authors additionally study the relation between Rab10 and Dystroglycan/Dystrophin (Dystrophin-associated protein complex, DAPC), which they previously showed to be essential for fibril formation (Cerqueira-Campos et al., 2020). They show here that Dystrophin and Rab10 colocalize at the basal trailing side of follicle cells and that overexpressed Dystroglycan can recruit Rab10 to the plasma membrane; however, they also show that Dystrophin mutants fail to display an effect on Rab10 localization, leaving the significance of the proposed Rab10-DAPC interaction unresolved. Finally, the authors present convincing evidence that the exocyst complex opposes fibril formation, and suggestive but comparatively weaker results pointing that this opposition is due to two independent separate exocyst roles: an inhibitory interaction exocyst-Dystrophin (Dystrophin being required for fibril formation), and a positive role in the alternative Rab8 non-fibril route.

Major comment:

- There are several instances throughout the study in which the authors seem to have problems quantifying results. This affects some assertions central to the message of the paper that are not supported by the quantifications presented. It also casts doubts on accessory points deduced from quantitative differences (or lack of difference) that do not seem fully reliable. I would urge the authors to reevaluate their quantification methods.

a) Rab8 KD does not significantly increase apical fraction of Collagen IV with respect to control (Fig. 1H). The image in 1C clearly shows that Col IV is present apically, something that has been shown by others and that never occurs in the wild type. Failure of the quantifying method to detect a difference can only mean the quantifying method is not adequate. A 10% average in the control when it's clear that no Col IV at all is found apically in the wild type suggests that the authors are quantifying background signal that they should not be acquiring, and, if acquired, they should be subtracting. Rab8/Rab10 double knock down is said to show a synergistic effect, when an additive effect would be more consistent with alternative routes. Other problematic deductions drawn from apical fraction quantifications are

found in Fig. 5J (Dys- enhancing Rab8 KD but not Rab10 KD) and Fig. 7D (Exo70- enhancing Rab10 KD but not Rab8 KD).

We agree that this quantification was not optimal. We improved it by quantifying a narrower and more precise region for each domain. The new results are shown in Figure 1H. This improvement reduces the apical signal in the control from 10% to 6% and allows us to detect a significant increase between the control and Rab8 KD, thus resolving the problem raised. After verification, we did not subtract the background because there was no electronic background in our images (i.e. black is really black and equal to zero). Thus, the remaining signal is the true cytoplasmic GFP signal and it may not be appropriate to subtract it. Other data (fig 5J and 7D, now named fig 5G and 7H) were also re-analyzed with no major change.

b) Similar to apical fraction, measurements of planar polarization (trailing/lateral ratio) show average ratios near 1 for Dg, Rab10 and Dys, which is striking given that the localization of these proteins is so clearly polarized. Ratios lower than 1, which are reported for many individual cells in these graphs, should mean reversed polarity. In light of this, I would not be too confident on the effects reported in 5O-Q. In fact, on two occasions, the authors obtain significant differences in these planar polarization measurements that they themselves disregard: Fig. 6J (Rab10 in Exo70-) and Fig. 7I (Dys in Rab8 KD).

We agree that this quantification could be improved. Our initial quantification of the planar polarized proteins, Rab10 and Dys, found at the trailing edge, was confounded by their lateral spread. We have now reported with only the front half of the lateral side. By doing this for instance on Figure 5, we increased the ratio in the control conditions, with almost no points below the value of 1, while the conditions in which polarity is visually affected are unchanged and still close to 1. Thus, this new quantitative approach reinforced our conclusions on this figure.

For the figure 6, this new analysis confirm our previous observations : we observed a significant effect of Exo70 mutant, but not of Exo70 overexpression, on Rab10 localization (Figure 6J) while both impact Dys localization (Figure 6F). Main text mentions these two results.

Regarding the effect of Rab8 on Dys localization, we indeed observed a slight decrease of its polarization that we currently cannot explain (Figure 7). The important point here is that this effect is opposite to the one observed in Exo70 mutants. Thus, Exo70 effect on Dys cannot be explained by the fact that Rab8 route is blocked in this context. Text has been modified: "Conversely, Rab8 KD *slightly affected Dys localization, but, importantly, this effect is opposite to the one observed in the* Exo70 *null mutant (Figure 7I)."*

c) Quantifications of lateral fraction Col IV in mosaic experiments do not support decreased lateral secretion in Rab8 OE (3G) or Dys- (S5C), which are central tenets of the study.

We have endeavoured to detect such differences in Dys mutants and Rab8 OE and do not see any possible improvement in the quantification method and we therefore attempted, instead, additional experiments.

With respect to Rab8 OE, we suspect that this gain of function is not sufficiently effective under the specific conditions of the experimental setup described in Figure 3, as its effect appears to be more subtle than that of Rab10 OE in Figure 2. We therefore tried to repeat this experiment in a sensitized background in which Rab10 function was partially affected. Unfortunately, we did not see an improvement. However, since downregulation of Rab10 is not sufficient on its own to induce significant differences in this experimental setup, such an experiment is unconclusive and was not added the article. Nonetheless, we modified the results and the discussion to underly the data we got that strongly support that Rab8 route is targeted towards the basal domain with for instance the fact that Exo70 is required for Rab8 route and for basal secretion of collagen.

Regarding Dystrophin, we attempted to see whether its effect could be specific on its canonical ECM ligand that is Laminin A. Though we did not have the proper construct (UAS:LanA-GFP) to reproduce the same experiment set-up as with collagen, we tried to see whether Dg overexpressing clones, in presence or absence of Dys, were able to target LanA-GFP(under its own promoter) to the lateral domain of the cells. However, the result was negative and the experiment has not been included in the article. Thus, potential explanations of our results involving Dystrophin and Dystroglycan are detailed in the discussion.

Minor comments:

- It is stated that Rab10 and Dys associate with tubular endosomes, but no data here support identification as endosomes of these tubular structures, to my understanding.
We agree with this comment and we modified the text accordingly, mentioning a "tubular compartment" or" a subcellular compartment, with structures reminiscent of tubular endosomes."

- The authors call sup-basal the cell region immediately apical to the most basal. Is there sufficient reason to not call this lateral? If a new term is needed, shouldn't it be supra-basal?
It was changed everywhere for supra-basal.

- In Fig. S1A and B, Col IV is labeled as green but represented in cyan.
Sorry for this mistake that has been corrected.

- Fig. S1A should present a wild type control.
A control has been added.

- It is not clear where Y2H results in Fig 6A come from.
The Legend has been modified to make it clearer : " scheme of Dys domains and the fragments identified in a yeast two-hybrid screen with Exo70 as prey (Formstecher et al, 2005)."

- Fig. 3C'-E' label suggests a gradient made from multiple images, but it looks like just two images and two colors.
It is actually a true color gradient depending on z axis but the signal is indeed mainly found at the extremities of the z-stack.
- Graphs in Fig. 3H-J, S5D and 7B are not legible.
Graphs have been improved.

- Fig. S1B does not seem to make a significant point in the context of this study.
Although we understand this comment, we followed suggestion of R#2 who asked in its major comments for more details with other cell polarity markers.

- I suggest drawing a summary scheme to aid readers better assess interpretations alternative to the ones given in the text.
Such a summary scheme is now shown in the last figure.

Reviewer #1 (Significance (Required)):

This study reports important new information on the secretion of Collagen IV by polarized cells of the Drosophila follicular epithelium. It complements previous studies on the roles of Rab8, Rab10 and Dystroglycan/Dystrophin, additionally uncovering a role for the exocyst complex. Addressing some issues with quantitative imaging should increase confidence in its most critical conclusions.

Reviewer #2 (Evidence, reproducibility and clarity (Required)):

Summary:
In this manuscript, Dennis et al. identify different secretory routes and cell exit sites involved in basement membrane secretion and diversification in epithelial cells. Using the follicular epithelium of the Drosophila ovary as their model system coupled with genetics, imaging, and image analysis approaches, they show that two previously identified RabGTPases, Rab8 and Rab10, work in parallel routes for basement membrane secretion. These two small GTPases work in a partially redundant manner, where Rab8 promotes basal secretion leading to a homogenous

basement membrane, while Rab10 promotes lateral and planer-polarized secretion, leading to the formation of fibrils. The authors also show that Rab10 and the dystrophin-associated protein act together to regulate lateral secretion, and dystrophin (Dys) is necessary for dystroglycan (Dg) to recruit Rab10. Furthermore, DAPC is shown to be essential for fibril formation and is sufficient to reorient Collagen IV to the Rab10-dependent secretory route. Dys was also shown to interact directly with exocyst subunit Exo70. Using overexpression and loss of function approaches the authors claim that Exo70 limits the planer polarization of Dys, and as a result, Rab10, hence limiting basement membrane fibril formation. Finally, the authors state that the Exocyst (Exo70) is also required for the Rab8-dependent basement membrane route. Overall, the data described in this manuscript are convincing and the authors' claims are supported by the presented data. We have mainly minor comments and only a few major comments that need to be addressed.

Major Comments:

• In the text for Figure 1G-H (page 4), the authors stated that the basal secretion was not restored in Rab8, 10, and 11 triple KD, in our opinion, it is unclear how the authors came to this strong conclusion from the presented data. It would be good if the authors explicitly explain how they come to this conclusion. Is it only based on the weak Coll-IV-GFP signal in the Rab8, 10, and 11 triple KD data compare to the control? If so, the authors should statistically quantify the difference with the control. In Figure 1H, no statistical analysis is provided between the control and triple KD conditions.

We agree that it was not entirely appropriate to give such conclusions on the basis of the quantifications available. A new graph showing basal fluorescence intensity (new Figure 1H) (and not just the ratio of apical to apical plus basal as in Figure 1I) has been added to better support the text. A relevant statistical comparison has been added to Figure 1H (old Figure 1I). We apologize for this oversight.

• From the data presented in Figure S1B, the authors state that the basement membrane mislocalization observed in Rab8/10KD has no major impact on polarity maintenance. They based this statement only on the localization of the apical marker aPKC. Although the aPKC data are convincing, it would be more compelling if the authors observe the distribution of other polarity proteins such as Dlg, E-Cadherin, and armadillo to better assess if the overall epithelial polarity is maintained in this condition.

Dlg and Ecad staining in these different genotypes were added to figure S1 with no major impact on the conclusions.

Minor Comments:

General comments:

• In the text describing their data, we recommend that the authors clearly indicate which panel(s) they are referring to.

We paid attention to this point in the revised manuscript.

• The authors should also be consistent with the diction throughout the manuscript when referring to the cortical domain or region of the cell (back/rear/trailing edge/leading edge).

We tried to be more consistent. We now only speak about the "front" for one side and for the other of "trailing edge" or "rear half of the cell", the latter corresponding to a more extended part of the cell than the previous. A scheme on figure 2A illustrates these terms.

The following specific comments are in order of appearance in the manuscript.

Introduction Section:

The following statements in the introduction should be supported by specific references:

• "BM is critical for tissue development, homeostasis and regeneration, as exemplified in humans by its implication in many congenital and chronic disorders."

We added the following reference: (Sekiguchi and Yamada, 2018)

• "BM is assembled from core components conserved throughout evolution: type IV collagen (Col IV), the heparan sulfate proteoglycan perlecan, and the glycoproteins laminin and nidogen."
We added the following reference: (Mouw et al, 2014)

• "During development, the dynamic interplay between cells and BM participates in sculpting organs and maintaining their shape."
We added the following references: (Sherwood, 2021; Jayadev and Sherwood, 2017; Walma and Yamada, 2020; Pastor-Pareja, 2020).

• "BM protein secretion shows some specificities, mainly because of the large size of the protein complexes (e.g., procollagen) that must transit from the endoplasmic reticulum to the cell surface". This statement could be supported with references including specific Drosophila references. Additionally, the authors need to clarify what they mean by "some specifies".
We added the following references: (Ke et al ,2018; Feng et al, 2021).

Results section:
• In the text describing Fig. 2 (page 5), the authors describe two different basement membrane types: fibrils and homogenous. Moreover, the manuscript focuses on the role of Rab8 and Rab10 in the formation of these two structures. Thus, the authors must better describe the two different types of basement membrane structures and their known roles. This will be helpful for the readers to analyze the presented data, especially for those that are not familiar with the system.
We rewrite the beginning of this paragraph : " Follicle BM is composed of an homogenous matrix from the very first stages while BM fibrils are added during the collective cell migration (Figure 2A, top) (Haigo and Bilder, 2011; Isabella 2016). Although the exact contribution of each of these BM types is not yet fully understood, genetic manipulation indicated that they are both required for the proper morphogenesis of the future egg (Haigo and Bilder, 2011; Isabella et al, 2016; Cerqueira Campos et al, 2020). Findings mainly based on gain of function experiments suggest that Rab10 participates in the follicle cell BM diversification by contributing to the formation of BM fibrils that are deposited as the cells migrate (Isabella et al, 2016). On the other hand, the route to generate homogenous BM remains unknown."

In Figure 2A, the authors describe stage 3 basement membrane as uniform BM, do they mean homogenous?
Figure 2A has been corrected.

• In the text describing the data for Fig. 3 (page 6), the authors should clearly explain the reason to use anti-GFP antibodies in a non-permeabilized condition (i.e., to detect specifically the extracellular secretion of BM proteins). This will help the readers to interpret the data presented.
It is now explained as following " Thus, detection of Col IV with an anti-GFP antibody and a Cy3- or Cy5-conjugated secondary antibody without permeabilization allowed discriminating secreted collagen from the total protein."

• On page 9, the authors stated that the precise localization of Dg in follicle cells is unknown. This statement is incorrect. It has been shown, using a Dg antibody, that Dg localizes at a high level at the basal side of the follicle cells and at a lower level at the apical side (Deng et al, 2003 and Denef et al. 2008).
It has been corrected : "Endogenous Dg was described by immunostaining to be mainly enriched on the basal side of the cells (Denef et al, 2008; Deng et al, 2003)."

Discussion Section:
• The following statement is not clear: "Thus, three different Rab proteins are targeted towards the three distinct domains of epithelial cells defined by apical basal polarity, and at least of them is also planar polarized". The authors should rephrase and describe specifically which Rabs they are talking about.

Text has been changed as following "Thus, these three different Rab proteins, Rab11, Rab10 and Rab8, are targeted towards the three distinct domains of epithelial cells defined by apical basal polarity, apical, lateral and basal, respectively. "

• This statement is vague: "These three Rab GTPases have been jointly involved in different processes (Knödler et al, 2010; Sato et al, 2014; Vogel et al, 2015; Eguchi et al, 2018; Häsler et al, 2020)". The authors could also mention the processes in which Rab8, 10, and 11 are involved.
We tried to be more precise : "The same three Rab GTPases have been jointly involved in different processes such as ciliogenesis, targeted exocytosis or lysosome homeostasis where they have been proposed to act in a redundant manner"

• The following statements need to be supported by references. "Therefore, more investigations are required to define exactly how the DAPC allows the formation of BM fibrils. Nonetheless, given the importance of the DAPC and BM proteins in muscular dystrophies, our results will pave the way to determine whether a similar function is present also in muscle cells. Interestingly, the extracellular matrix is different between the myotendinous junction and the interjunctional sarcolemmal basement membrane and may provide another developmental context where several routes targeted to different subcellular domains may be implicated".
The following reference has been added : (Jacobson et al, 2020).

Experimental Procedure Section:
• In the dissection and immunostaining section (p14), there is a typo: it should be for "20 min" instead of "2for 0 min"
It has been corrected.
• For the GST pulldown experiments, the authors mention that they use a standard protocol to produce S35 Exo 70 and the GST pulldown experiments. The authors should provide references.
A reference has been added.

Figure and Figure Legend:
• General comment: The orientation of the images showing the rotation and leading and trailing edges need to be consistent in the different figures (e.g., In Figures 3 and 7, the leading edge is oriented to the top while in Figures 4, S4, 5, 6, the leading edge is oriented to the bottom). This will help the readers to analyze the data.
We apologize for this, and we carefully checked image orientation throughout the figures.

• In Figure 1 C-G the scale bars are missing and should be added as Fig. 1B. • In Figure 4, some scale bars are missing. • In Figure 6, some scale bars are missing.
Scale bars have been added.

• Figure S1A: The data presented in Figure S1A is convincing. However, a control panel should be added showing the absence of apical Coll IV for comparison. This information will help with the interpretation of the data.
A control has been added.

• In Figure 3 legend: it should be "immunostained" for GFP instead of stain for f-actin and GFP. • In Figure 4 legend: it should be "(A, E)" after (i.e 0.8 μm above the basal surface) instead of "(C, G)". In Figure 5 legend (p23), it should be "plane" and not "plan".
Legends of figure 3, 4, 5 have been corrected.

• In Figure 5A-E, the authors show quantification of the fibril fraction for Dys-, Rab10 OE, and Rab10OE+Dys, Rab8KD, and Rab8KD+Dys-, and images of the collagen fibril for all the conditions except Dys-, it will be informative that the authors present a representative image of the Coll IV fibril in Dys- condition for comparison. The above comment also applies to Figure 5F-J, and it will be also informative to have a representative image of Dys- condition.
The requested panels have been added.

• Overall, the legend for Fig. S5 is not clear and we recommend the authors to clearly described the different panels. (e.g., it should be "(D)" instead of "(H-J)")
Legend is now detailed as requested.

Reviewer #2 (Significance):

Despite the important roles of the basement membrane for mechanical support, tissue and organ development, and function, the mechanisms that control the polarized deposition of basement membrane proteins are largely unknown. The contribution of Rab 8 and Rab 10 in the polarized deposition of the basement membrane was previously shown. However, by identifying two competitive secretory routes for the basal secretion of the basement membrane proteins that required these two different RabGTPases, controlled by the DAPC and the exocyst complexes, the authors make a novel contribution to our understanding of the mechanism that leads to the polarized secretion of basement membrane proteins (in that case Collagen IV). Since the basement membrane has critical roles in tissue and organ morphogenesis and functions, and its misregulation has been associated with developmental defects and pathological conditions, this research sheds light on the mechanisms important in these morphogenetic processes and will give insights into their deregulations in pathological conditions.

Reviewer #3 (Evidence, reproducibility and clarity):

In the present work the authors have elucidated a novel mechanistic model of basement membrane morphogenesis using Drosophila ovarian follicle cells as a model. The authors have employed extensive quantification approaches to justify the spatio-temporal expression of the molecules under study such as Collagen IV, Rab8, Rab10, DAPC, etc. The authors suggest distinct exit domains of BM protein Collagen IV facilitated by Rab8 and Rab10 via distinct routes as they interact with each other. The authors further show that DAPC plays an essential role in Rab10 mediated baso-lateral fibrillar BM synthesis whereas Rab8 functions are more Exocyst (Exo-70 dependent).

Major comments:
Result 1:
The authors use RNAi lines to arrive at their conclusions, however, the extent of inhibition of gene expression achieved by the RNAi, has not been justified. Also observations from only one RNAi stock may not be completely conclusive:

i) Efficiency of RNAi has not been tested or shown. No supporting data.
Rab10-RNAi stock is 26289 BDSC which is in Valium10, which is a weak RNAi line and needs a Dicer.
ii) Can same observations be made using classic alleles or generate somatic clones on follicular epithelial cells?
R#3 raised several questions regarding the efficiency of RNAi, the use of different lines and/or the use of classical mutants as an alternative method.
For Rab10, we tested three different lines with similar results as shown now in Figure S1A-B. These data are also consistent with those obtained by overexpression of a dominant-negative form of Rab10 (Lerner et al, 2013). Unfortunately, Rab10 is located extremely close to the X chromosome centromere and is even more proximal than the FRT transgenes. It is therefore impossible to generate somatic mutant clones.
Regarding Rab8, it is already published that Rab8 RNAi, expression of a dominant-negative form of Rab8 and Rab8 mutant cells obtained by somatic clones give similar defects (Devergne et al, 2017). The text has been modified to better illustrate the available data validating our approach.
In addition, mutant clones would not allow analysis of genetic interactions in complex genetic contexts such as double and triple KDs. Similarly, the choice of the Rab10 line was motivated by the ease of obtaining the appropriate genetic combination according to their genomic location.

iii) Intensity of Collagen IV in the basement membrane in Rab11 knock-down mutants seems to be significantly low

as compared to the Rab8 and Rab10 knock downs in supplementary Fig 1B. Are the authors very sure that Rab11 has no functions in basement membrane basal organization?
Good catch! Indeed, Rab11 RNAi significantly reduces basal secretion as now shown on fig 1H. Rab11 has pleiotropic functions in epithelial cells notably for their polarity (Choubey and Roy, 2017, Fletcher et al, 2012… and Fig S1). Thus, the reason for such a decrease is unclear and could be an indirect consequence of an overall abnormal epithelial structure. Thus, we now report this observation but have not taken its interpretation too far.

iv) Authors need to show where and how fluorescence intensities have been measured.
Magenta rectangles with dashed lines on Figure 1A illustrate the ROIs used for this analysis and more details have been added in the 'experimental procedures' section.

Result 2:
Confusing diagram. The authors should clarify whether the BM fibrils indicate lateral or planar BM components which they show to be more prominently expressed in Rab10 over-expression mutants.
A short note or an accompanying explanatory diagram on the source of the BM fibrils in the cellular context should make things less confusing.
Schemes on figure 2A have been improved to make it clearer.

FF calculation is an ingenious way of trying to look into functions.

The term Opposite effects/functions may be reconsidered as Rab8 and Rab10 compete with each other to deposit Collagen at spatially distinct domains. Opposite functions may give an impression that Rab8 actually represses Rab10 activity or vice versa, which may not be the case here.
Text has been modified as suggested, speaking about "contrary effects" and "distinct functions".

Result 3:
Why was anti-GFP Ab detected with Cy3-Cy5 secondary Ab. GFP itself is green so why detect it with a Red secondary? Logic? How clone Collagen GFP and ECM collagen GFP was differentiated? Please justify
It is now explained as following " Thus, detection of Col IV with an anti-GFP antibody and a Cy3- or Cy5-conjugated secondary antibody ithout permeabilization allowed discriminating secreted collagen from the total protein."

A panel with a dotted line joining the peripheral or lateral Collagen as shown in panels D' E' of Fig 3 would support the cartoon provided and link the cartoon to the actual microscopic images.
Figure has been modified as suggested.

Result4:
The authors suggest a UAS-Rab10-RFP transgene show same results as endogenous Rab10-YFP as compared to spatial expression pattern. This is worrisome as expression of full length functional gene tagged with a fluorophore may be an overexpression. A control experiment would be helpful in suggesting/comparing with the Rab10 OE phenotype and that will be more convincing.
We are not sure that we fully understand the reviewer's comment. However, we initially compared endogenous Rab10 and UAS-RAB10 at 25°C, a temperature at which the latter has no visible impact on BM structure (Cerqueira-Campos et al, 2020). Furthermore, even when higher expression was induced (by increasing the temperature and therefore Gal4 activity) and this had an impact on BM structure, this did not change the subcellular localization of Rab10, i.e. it was still planarly polarized, as shown in Fig 5S. The text has been modified to emphasize this point.

When the authors mention back of cells, where do the authors exactly mean? A cartoon of "the back of follicle cells", wrt the entire ovarian follicle would be helpful.
As asked by R#2, we are now more consistent throughout the paper, and a scheme illustrates these terms on Fig 2A.

The authors suggest basal Rab10 expression domain near the Golgi exit point. Can the authors use a Trans-Golgi marker in order to confirm this statement other than the references stated?
Trans golgi marker and Rab10 are now shown on figure S3.

Result 5
The authors may provide a Rab10 expression profile in DAPC null or KD mutants which would make their claims more comprehensive.
Data showing Rab10 localization in Dys mutant cells are shown on Figure S4A-B.

Result 6:
Exo 70 is a versatile molecule and Rho kinases such as Cdc42 can direct Polarised exocytosis through interaction of Rab effectors with Exo 70. Have the authors considered this?
We agree that it is an interesting prospect, but we consider it as beyond the scope of this article.

In general some immunostainings should be carried out if not in all at least in some experiments with some cell domain specific markers, more specifically PCP markers such as Flamingo/Vangl and basolateral markers such as Lgl/Dlg. This makes the positions specific claims of the authors more valid in the eyes of the reader.
We agree that this may help the reader but the mentioned pcp markers are not expressed in this tissue. However, the tissue planar orientation is now systematically indicated and consistent in all figures. We did not generally perform immunostaining for lateral markers but routinely included F-actin staining to detect cellular cortex. Our quantifications or cortical segmentations were based on the cell outline provided by this stain. On the basis of this staining, the outline of the cells was added on certain figures to facilitate understanding of the images.

Reviewer #3 (Significance):
The findings impinge on a critical cellular process of Rab protein interactions in the genesis of the basement membrane which is of potential interest. This falls under basic research. Since Rab molecules have emerged as molecules governing membrane morphogenesis, Cell and Molecular Biologists as well as a wide audience including clinicians will be interested on this.
